# Aseptic inflammatory abscesses induced by crizotinib in a case report of ALK rearrangement lung adenocarcinoma

**DOI:** 10.3389/fonc.2026.1812641

**Published:** 2026-05-14

**Authors:** Hongyin Huang, Keke Wang, Xu Zhai, Pu Cheng, Haihong Li, Wen Qiu, Tong Peng, Leilei Cao, Qizhi Luo, Zilong Wang

**Affiliations:** Department of Burns and Plastic Surgery, The Seventh Affiliated Hospital, Sun Yat-sen University, Shenzhen, China

**Keywords:** adverse events, aseptic inflammatory abscesses, case report, crizotinib, lung carcinoma

## Abstract

**Background:**

Adverse events induced by crizotinib treatment for lung adenocarcinoma are increasingly reported, with nephritic inflammatory abscesses accounting for only 4% of documented cases. To date, no reports exist regarding aseptic inflammatory abscesses of the back. Consequently, these conditions are often misdiagnosed clinically as cancer metastases or other infections, leading to delays in patient treatment. We report a case of a 34-year-old female patient who developed aseptic inflammatory abscesses in the back and nephritic inflammatory abscesses induced by crizotinib treatment for ALK-rearranged lung adenocarcinoma. The inflammatory abscesses improved after discontinuation of crizotinib. Subsequently, the patient received reduced-dose crizotinib therapy, with follow-up showing reduced complications and satisfactory tumor control. This report aims to fill gaps in the understanding of crizotinib-related adverse events, thereby enhancing the accuracy of diagnosis and management of these adverse events.

**Case report:**

A 34-year-old female patient with stage IVB right lung adenocarcinoma, accompanied by lymph node, liver, and bone metastases, received crizotinib therapy. After two years of crizotinib treatment, imaging studies revealed an aseptic inflammatory abscess in the back and left kidney. The lesions in the back and left kidney continued to enlarge despite incision and drainage, and the back abscess underwent surgical excision one month later. Pathology revealed aseptic inflammatory reaction without cancer cells. However, the postoperative back incision healed poorly with persistent purulent discharge, and the nephritic abscess continued to enlarge. Subsequently, based on radiographic and pathological findings suggestive of crizotinib-related adverse effects, the dosage of crizotinib was discontinued. The aseptic inflammatory abscess on the back rapidly improved after crizotinib discontinuation. The nephritic abscess, however, developed infection post-discontinuation and significantly reduced in size following antimicrobial therapy and drainage. Subsequently, crizotinib dosage reduction was implemented based on genetic testing. During follow-up, the inflammatory abscess continued to shrink without compromising tumor control efficacy.

**Conclusion:**

Early identification and diagnosis of crizotinib-associated aseptic inflammatory abscesses are critical. During treatment, reducing crizotinib dosage does not lead to recurrence of lung cancer while minimizing complications. The case underscores the necessity of integrating imaging and pathological features in the diagnostic process, alongside the need for personalized adjustments and development of treatment plans tailored to each patient.

## 1 Introduction

Lung adenocarcinoma primarily originates from the bronchial mucosal epithelium and mucous glands, accounting for 40% to 50% of all lung cancers and representing the most common type of lung cancer (1). Crizotinib is a first-generation ALK tyrosine kinase inhibitor (ALK-TKI) (2). Studies indicate that patients with advanced ALK-positive lung adenocarcinoma (3) experience effective control and alleviation of tumor progression and symptoms following crizotinib treatment (4). However, despite the promising effects of crizotinib, adverse events cannot be overlooked.

An increasing number of crizotinib adverse events have been reported: ranging from common gastrointestinal symptoms and hematologic side effects to rarer toxicities such as crizotinib-associated nephritic abscess and multi-organ aseptic abscesses (5–7). Among these reported adverse events, nephritic abscesses induced by crizotinib account for only 4%, while no reports currently exist regarding aseptic inflammatory abscesses of the back caused by crizotinib. When these rare adverse events occur, they are frequently mistaken for cancer progression or routine infections, thereby complicating patient diagnosis and treatment. Therefore, early recognition of crizotinib-associated adverse events is crucial.

This case report describes a patient with ALK-rearranged lung adenocarcinoma who developed aseptic inflammatory abscesses of the back and nephritic abscess after crizotinib therapy for two years. Following discontinuation of crizotinib, the aseptic inflammatory abscesses of the back resolved completely, and the nephritic abscess showed improvement. Subsequently, the patient received a reduced-dose crizotinib therapy, which resulted in decreased complications and maintained favorable tumor control during follow-up. This report documents a rare crizotinib-associated adverse event and aims to enhance diagnostic accuracy and clinical management.

## 2 Case presentation

### 2.1 Examination and surgical management of aseptic inflammatory abscesses of the back and nephritic abscess

**Figure 1:**
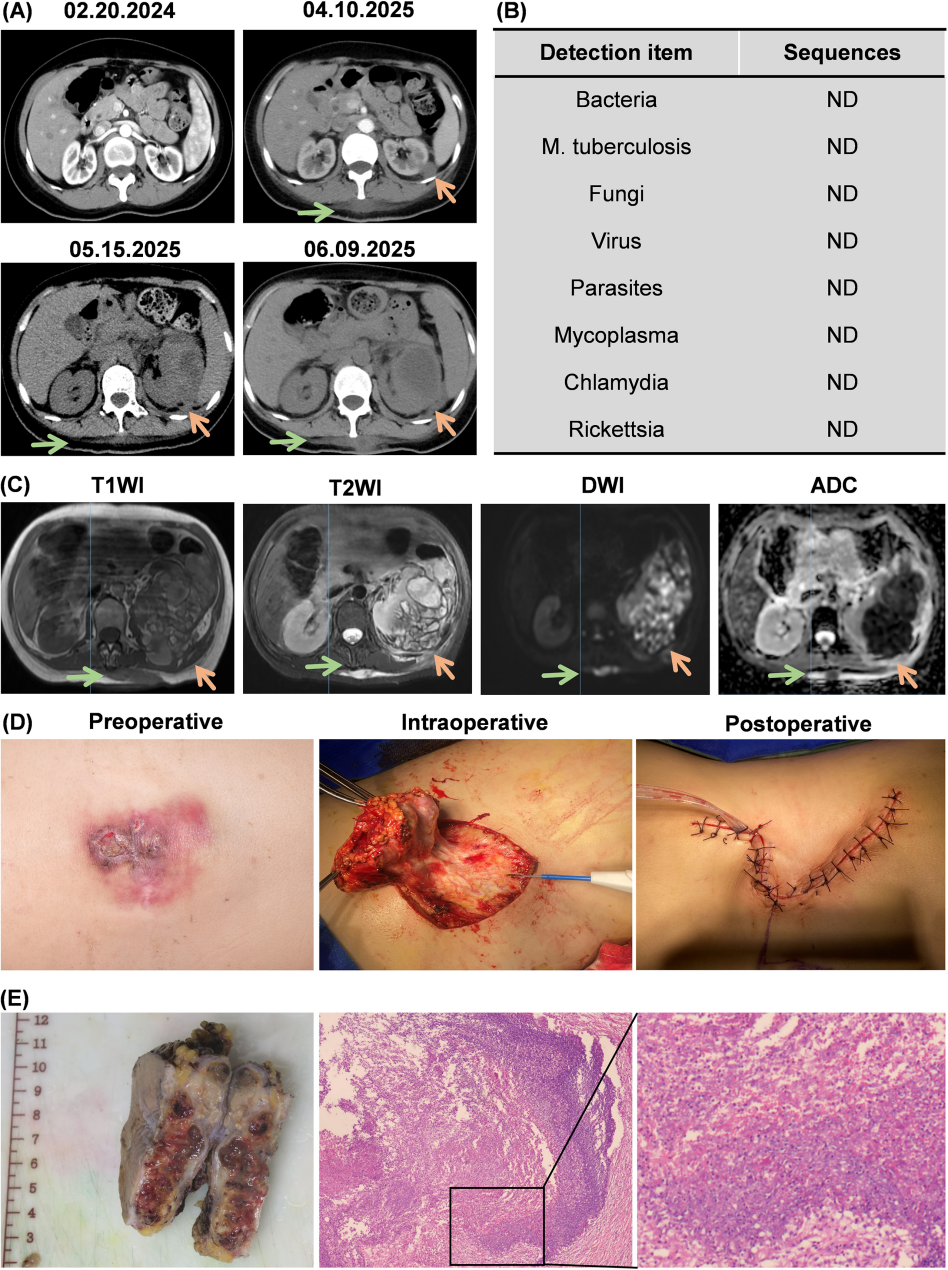
Examination and surgical management of aseptic inflammatory abscesses of the back and nephritic abscess. **(A)** abdominal continuous Computed Tomography (CT) Scan: February 20, 2024—no abnormalities in the back and the left kidney; April 10, 2024—initial detection of inflammatory abscesses of the back and nephritic abscess; May 15, 2024 - June 9, 2025: Progressive enlargement of the inflammatory abscesses in the back and nephritic abscess; **(B)** High-throughput microbial DNA testing of back puncture fluid: Negative results, indicating a aseptic inflammatory abscesses of the back; **(C)** July 2025 Magnetic Resonance Imaging (MRI) examination: The aseptic inflammatory abscesses of the back and nephritic abscess showed low signal intensity on T2-weighted images (T2W1) and high signal intensity on T1-weighted images (T1W1) within the cyst wall, along with diffusion-weighted imaging (DWI) showing diffusion suppression and corresponding apparent diffusion coefficient (ADC) mapping; **(D)** Preoperative, intraoperative, and postoperative findings of the aseptic inflammatory abscesses of the back. Intraoperative examination revealed a multilocular cystic abscess-like lesion. Complete surgical resection of the aseptic inflammatory abscess of the back region was achieved; **(E)** Pathological image of the aseptic inflammatory abscesses of the back: Chronic inflammatory changes were observed, with no malignant tumor cells present. ND, Not Detected; T1WI, T1-weighted imaging; T2WI, T2-weighted imaging; DWI, diffusion-weighted imaging; ADC, apparent diffusion coefficient.

A 34-year-old female patient was admitted in April 2023 for stage IVB(T3N3M1c2) right lung adenocarcinoma with lymph node, liver, and bone metastases. Molecular pathology analysis of the lung biopsy specimen revealed an ALK gene rearrangement. Targeted therapy was initiated in May 2023 with crizotinib 250 mg orally twice daily. Regular follow-up demonstrated excellent tumor control. One year after crizotinib therapy, CT scans showed no abnormal lesions in the back or the left kidney (**Figure 1A**). Hemoglobin, albumin, and lymphocyte percentage levels remained within normal ranges.

Two years after crizotinib therapy (April 10, 2025), the patient developed a subcutaneous abscess measuring approximately 5 cm × 3 cm in the back region of the back. The abscess gradually enlarged and began to discharge yellowish-white purulent fluid. Testing of the pus, blood, and urine for Mycobacterium tuberculosis DNA+RNA and acid-fast staining showed negative results. CT scans showed a septated, low-density nodule in the upper-middle portion of the left kidney, measuring approximately 2.2 × 1.9 cm with thickened walls, consistent with abscess formation (**Figure 1A**). Despite repeated incision and drainage and standard wound care, the back abscesses showed no improvement and continued to progressively enlarge. The nephritic abscess was managed conservatively. Follow-up upper abdominal CT scan showed the perirenal lesion enlarging to 4.2 cm × 5.1 cm (**Figure 1A**) one month later. Blood tests revealed: albumin 26.3 g/L; lymphocyte percentage 14.8%. On June 3, under local anesthesia, an ultrasound-guided percutaneous drainage of the left nephritic abscess was performed, obtaining 50 ml of bean-paste-colored purulent fluid. Postoperatively, the fluid underwent acid-fast staining, tuberculin gene testing, bacterial and fungal cultures, and high-throughput pathogen DNA gene detection. No pathogenic microorganisms were identified (**Figure 1B**). A follow-up CT scan one week later showed the left nephritic abscess had further expanded to 7.7 × 7.6 cm and was gradually encasing the middle and lower regions of the perinephric space (**Figure 1A**). The patient underwent ultrasound-guided percutaneous nephrostomy to drain the left nephritic abscess. The perinephritic drainage tube was removed after two days, and the patient was discharged.

One month postoperatively (July 18, 2025), the patient was readmitted due to the persistent and progressively enlarging aseptic inflammatory abscesses of the back. The affected skin area measured approximately 7×7 cm, exhibiting redness and swelling with ulceration. The back abscesses measuring about 10×8 cm, presenting with fluctuance. Bacterial and fungal cultures, along with tuberculin testing, were performed on the abscesses exudate, yielding no growth of bacteria or fungi. On July 22, 2025, MRI revealed (**Figure 1C**): Multiple cystic lesions at the T12-L4 level and the left nephritic region, with T1-low signal and T2-high signal walls. Diffuse restrictions were noted in the lesion areas, with surrounding patchy fluid accumulation. The left nephritic abscess measured 10.8 × 8.9 cm. The abscess area in the back had enlarged compared to previous findings, with a maximum cross-sectional dimension of approximately 10.2 × 8.3 cm. Follow-up blood tests showed: hemoglobin 66 g/L; albumin 29.6 g/L; lymphocyte percentage 17.2%. On July 24, 2025, the patient underwent excision of the back abscesses under general anesthesia (**Figure 1D**). Intraoperatively, the subcutaneous, multilocular cystic abscesses were visualized, containing yellow, gelatinous purulent secretions extending to the fascial surface (**Figure 1D**). Bacterial culture of the excised tissue revealed no bacterial or fungal growth. Histopathological examination demonstrated abundant foamy cells, lymphocytes, plasma cells, and neutrophils aggregated within the tissue, consistent with chronic inflammatory changes and no evidence of malignancy (**Figure 1E**). The patient was discharged on July 30, 2025.

### 2.2 Third hospitalization

**Figure 2:**
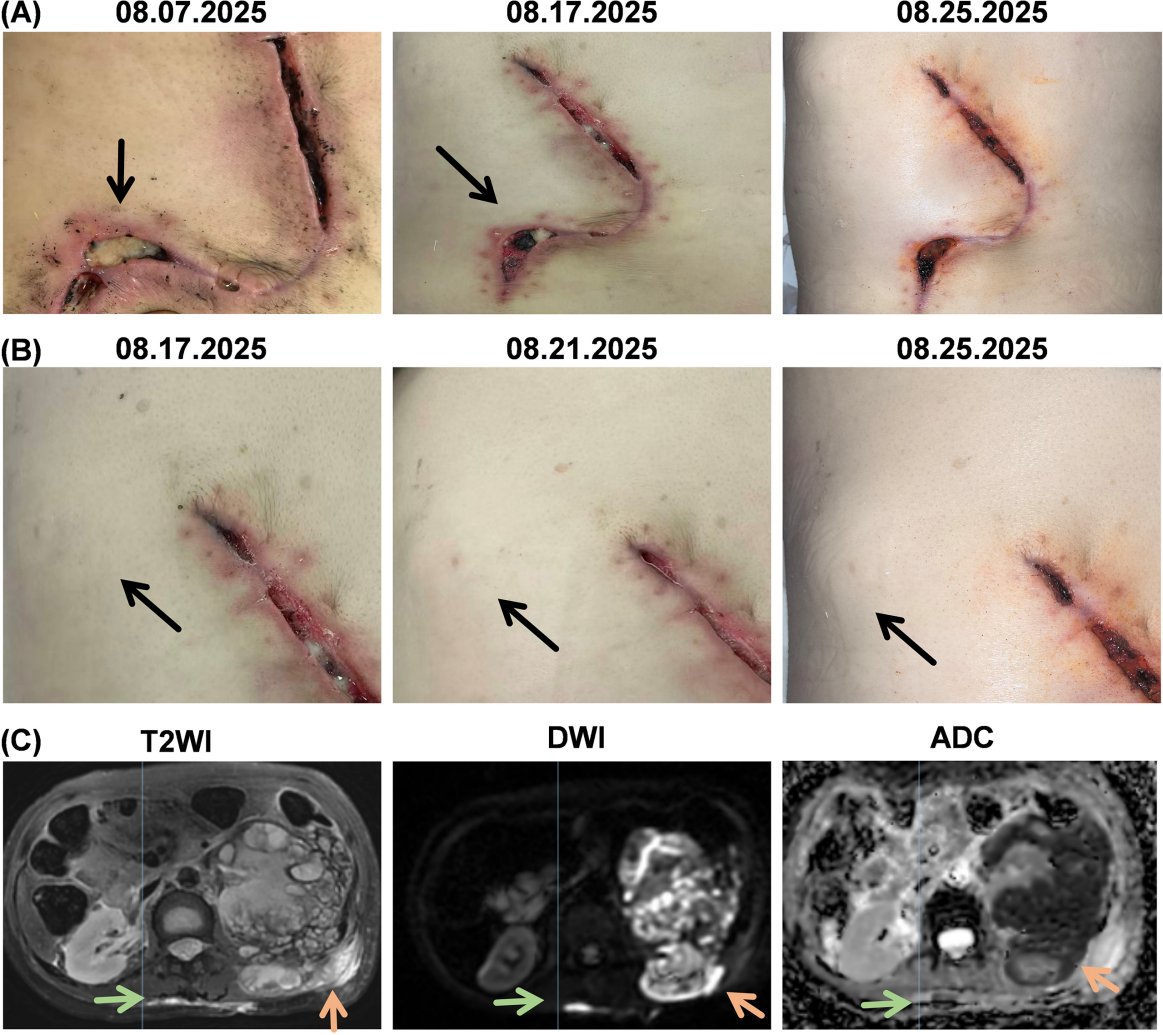
Back and nephritic lesions during third hospitalization. **(A)** 2025.08.07: Back incision dehiscence with persistent abscess-like fluid drainage. From 2025.08.17 to 2025.08.25, no significant improvement in incision condition following dressing changes; **(B)** 2025.08.17: First detection of a subcutaneous abscess at the 10 o’clock position of the back incision. The subcutaneous abscess gradually enlarged between 2025.08.21 and 2025.08.25; **(C)** 2025.08.24 MRI findings for aseptic inflammatory abscesses of the back and nephritic abscess: Low signal intensity on T2-weighted images (T2W1) within the cyst wall, diffusion suppression on diffusion-weighted imaging (DWI), and corresponding apparent diffusion coefficient (ADC) mapping.

One week postoperatively (August 7, 2025), the patient presented with wound dehiscence at the back surgical site, accompanied by yellowish, gelatinous exudate. Repeated dressing changes failed to improve the condition (**Figure 2A**). A new subcutaneous abscess measuring approximately 2 cm × 2 cm emerged at the 10 o’clock position of the incision (**Figure 2B**). MRI revealed further enlargement of the left nephritic abscess, with the largest transverse dimension reaching 12.4 × 12 cm (**Figure 2C**). During readmission, the back incision underwent wound healing therapies including ultrasound-assisted debridement yet showed no significant improvement in healing (**Figure 2A**). The new subcutaneous abscess at the 10 o’clock midline of the incision continued enlarging to 3cm x 3cm (**Figure 2B**). Complete blood count revealed: hemoglobin 63g/L; albumin 26.2g/L; lymphocyte percentage 14%. Yellow gelatinous discharge from the incision was cultured for bacteria, fungi, and tuberculosis with antimicrobial susceptibility testing, and no pathogenic microorganisms were detected. Pathological examination of the discharge revealed abundant mucus, a small number of histiocytes, lymphocytes, and neutrophils, with no tumor cells observed. Special stains: AB, PAS, hexamine silver stains were all negative; acid-fast staining was negative. This suggests no clear evidence of specific pathogen infection. Following a multidisciplinary consultation involving the Burn and Plastic Surgery Department, Oncology Department, Pharmacy Department, Infectious Diseases Department, Radiology Department, Urology Department, Pathology Department, Nephrology Department, and Laboratory Department, the back abscesses were considered the aseptic inflammatory abscesses, possibly an adverse event to crizotinib.

### 2.3 Discontinue crizotinib treatment

**Figure 3:**
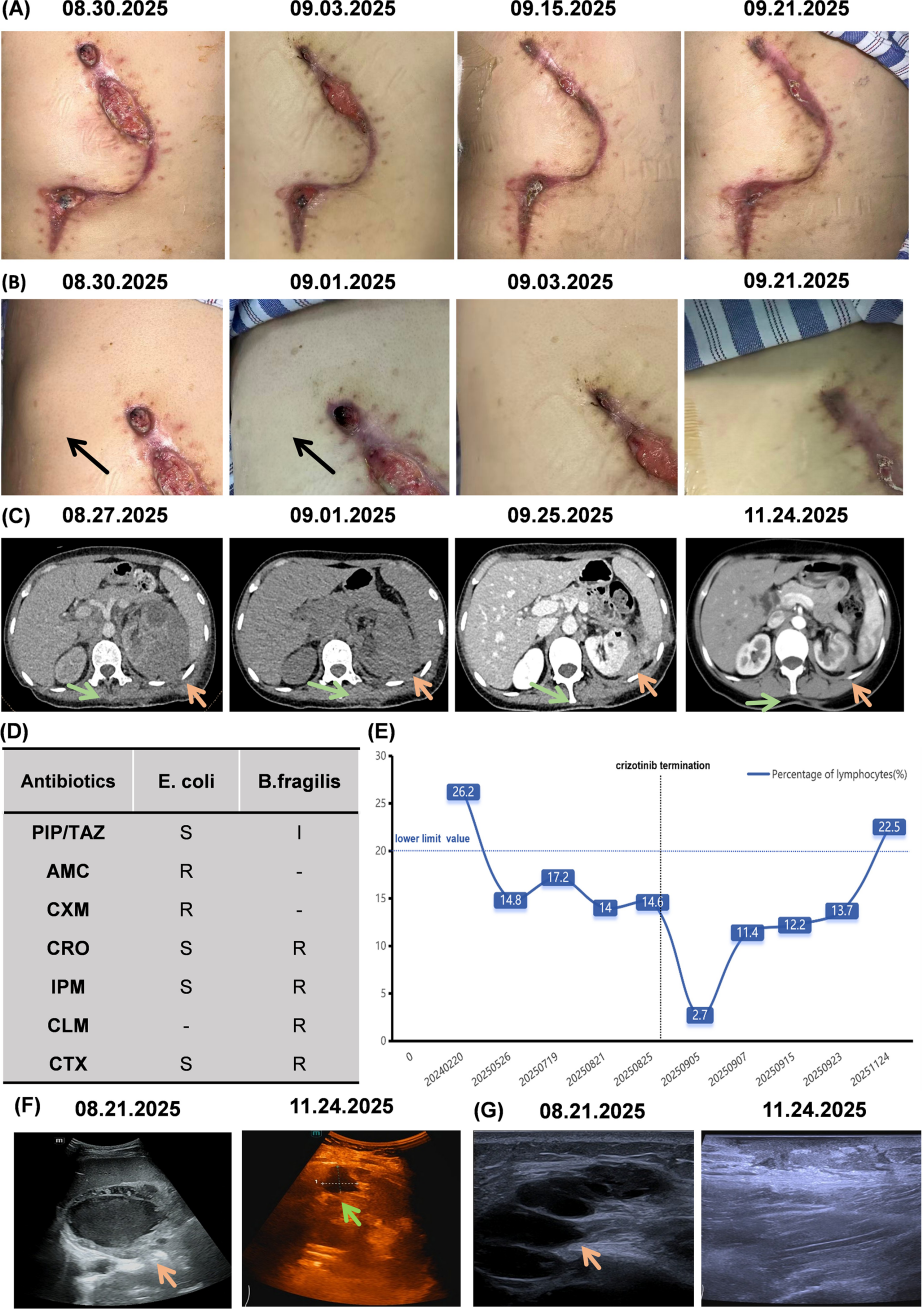
Cases of aseptic inflammatory abscesses of the back and nephritic abscesses following crizotinib-discontinuation and dose-reduction. **(A)** After discontinuing crizotinib, purulent discharge resolved and the back incision gradually healed. **(B)** Following crizotinib-discontinuation, the subcutaneous abscess at the 10 o’clock position of the back incision progressively diminished. **(C)** Abdominal CT: nephritic abscess gradually shrunk after crizotinib-discontinuation, anti-infective therapy and drainage. After the patient began tapering crizotinib, the nephritic abscess continued to shrink as of 2025.11.24; **(D)** Bacterial culture and sensitivity results from nephritic abscess drainage fluid and blood cultures: Escherichia coli and Bacteroides fragilis infection. Both organisms were susceptible to piperacillin-tazobactam; **(E)** Lymphocyte Percentage: Prior to the aseptic inflammatory abscesses detection, the patient’s lymphocyte percentage was within normal range. Following abscesses detection, the percentage fell below normal range. After discontinuing crizotinib, the lymphocyte percentage returned to normal range; **(F)** Color Doppler ultrasound findings: nephritic abscess prior to crizotinib-discontinuation on 2025.08.21; after crizotinib-discontinuation and dose reduction on 2025.11.24, the nephritic abscess showed significant reduction in size compared to previous assessment; **(G)** Color Doppler ultrasound findings: August 21, 2025 (pre-discontinuation): Aseptic inflammatory abscesses of the back. November 24, 2025 (post-discontinuation, after resuming crizotinib at reduced dosage): Aseptic inflammatory abscesses of the back had resolved. E. coli, Escherichia coli; B. fragili, Fragilis Bacterium; PIP/TAZ, Piperacillin/Tazobactam; AMC, Amoxicillin/Clavulanate; CXM, Cefuroxime; CRO, Ceftriaxone; IPM, Imipenem; CLM, Clindamycin; CTX, Cefotaxime.

On August 27, 2025, crizotinib was discontinued. The incision of the back aseptic inflammatory abscesses accelerated healing with resolution of discharge (**Figure 3A**). The subcutaneous abscess at the 10 o’clock position of the back incision also gradually diminished following the crizotinib-discontinuation (**Figure 3B**). Twenty-five days after crizotinib-discontinuation (September 21, 2025), the back incision had largely healed (**Figure 3A**), and the subcutaneous abscess had resolved (**Figure 3B**). During this period, follow-up blood tests showed gradual increases in hemoglobin and albumin levels. One week after crizotinib-discontinuation, a CT scan of the left nephritic abscess revealed a reduced size in the left nephritic region compared to previous scans (**Figure 3C**). the patient underwent a ultrasound-guided needle aspiration of the left nephritic abscess due to the pain in ipsilateral kidney. Two hours post- aspiration, the patient developed a fever of 39.3 °C (102.6 °F), suggestive of perinephric infection. Ceftriaxone 2 g was administered intravenously for antimicrobial therapy, but the fever persisted. Due to the infection, the patient’s lymphocyte percentage decreased to a low of 2.7%. Bacterial culture results after 4 days identified Escherichia coli and Bacteroides fragilis (**Figure 3D**). So the antibiotic was switched to piperacillin-tazobactam 4.5 g, leading to improvement in the fever. To eliminate the infection source, the patient underwent the catheter drainage procedures of the left nephritic abscesses on September 11 and September 17, targeting the lower and upper segments of the left kidney respectively. The drainage fluid was pyosanguinous. Cytopathological examination revealed numerous inflammatory cells and fibrinous exudate, with no evidence of malignancy. The patient experienced no further fever or pain. So the drainage tubes were removed 6 days later.

### 2.4 Adverse events and antitumor efficacy follow-up

**Figure 4:**
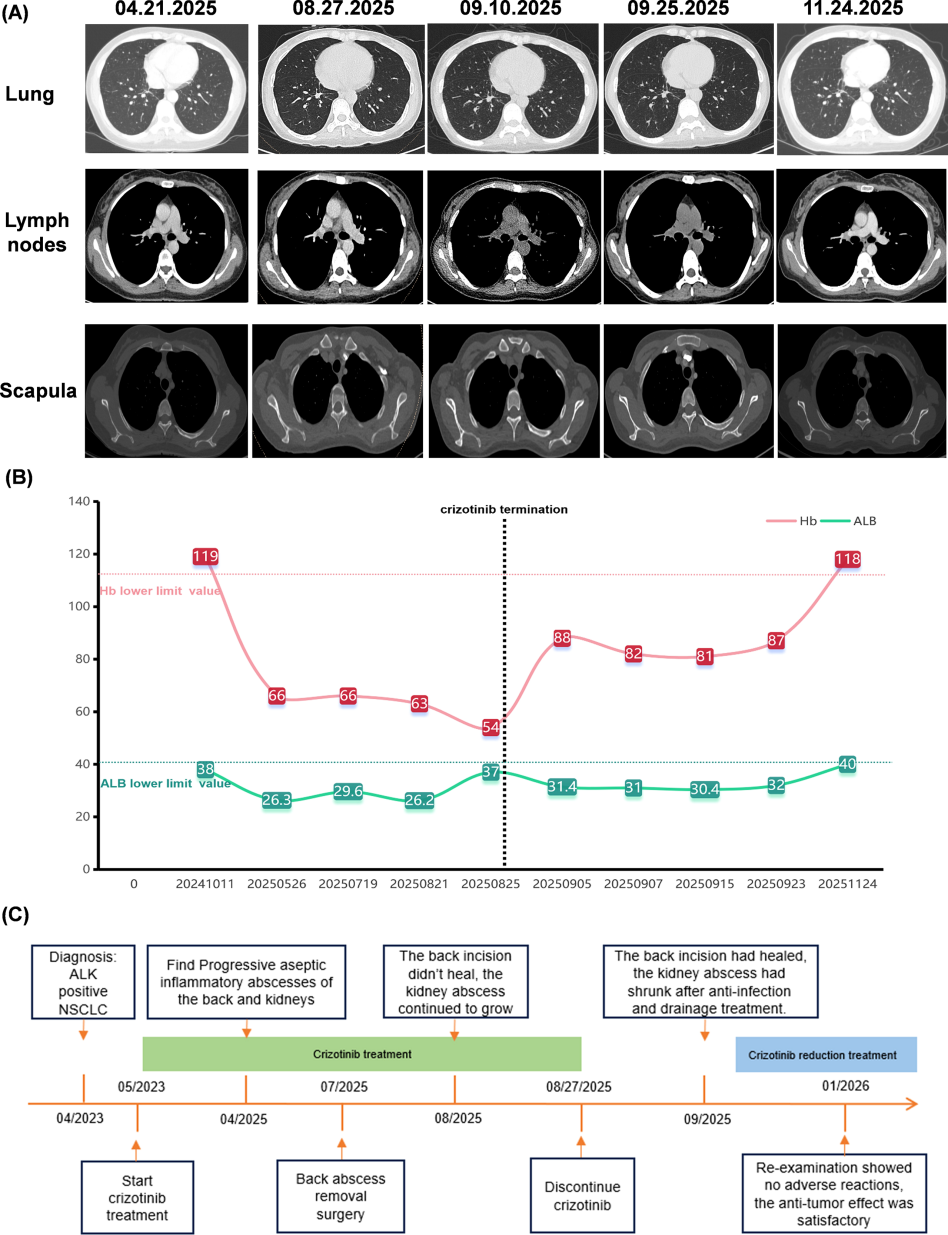
Patient tumor status, hemoglobin and albumin follow-up, and diagnostic and treatment flowchart. **(A)** Abdominal CT: Following crizotinib discontinuation and dose reduction, no progression was observed in metastatic lesions in the lungs, mediastinum, and scapulae. **(B)** Trends in hemoglobin and albumin levels: The patient exhibited low hemoglobin and low albumin during crizotinib treatment. Following crizotinib discontinuation and dose reduction, the patient’s hemoglobin and albumin levels gradually returned to normal. **(C)** Timeline diagram of the diagnostic and treatment process. Hb, Hemoglobin; ALB, Albumin.

On September 22, 2025, the patient was admitted to the Oncology Department for further refinement of tumor genetic testing. Blood count review: Hemoglobin 87 g/L; Albumin 32 g/L; Lymphocyte percentage 13.7%. A follow-up whole-body CT scan on September 25 showed the nephritic abscess had reduced to 4.1 × 3.9 cm (**Figure 3C**). Blood genetic testing indicated crizotinib sensitivity on October 2025. To prevent adverse events, the crizotinib dose was reduced to 200mg/day with regular monitoring to prevent recurrence. Ultrasound review from November 11–24 showed significant reduction of nephritic abscess (**Figure 3F**). CT scan showed the nephritic abscess had shrunk to 3.1×2.5cm (**Figure 3C**). Scar formation was observed at the surgical site on the back with no new abscess (**Figure 3G**). A blood test conducted on the same day showed complete recovery to normal ranges: lymphocyte percentage 22.5% (**Figure 3E**); hemoglobin 118 g/L; albumin 40 g/L (**Figure 4B**).

During the discontinuation or dose reduction of crizotinib, no significant changes were observed in the primary lung cancer lesion in the basal segment of the right lower lobe, multiple lymph nodes in the right hilar and mediastinal regions, irregular lesions at the bronchial origins, multiple solid nodules in both lungs and pleura, or high-density shadows in the right scapula and ilium (**Figure 4A**; **Supplementary Figures 1A, B**). Multiple hepatic metastases remained poorly defined, with only small calcifications visible in the right hepatic lobe (**Supplementary Figure 1B**). These findings indicated sustained anti-tumor efficacy without evidence of new metastases or emerging lesions. The diagnostic and treatment timeline is illustrated in **Figure 4C**.

## 3 Discussion

This case report describes a 34-year-old patient with lung adenocarcinoma who developed the aseptic inflammatory abscesses of the back and nephritic abscess as adverse events to crizotinib. After multiple examinations, imaging, pathological examinations, and surgery, tuberculosis infection and pathogenic infections were ruled out. Following verification of characteristic imaging and pathological findings, and through multidisciplinary discussion, the final diagnosis was established as the crizotinib-related adverse events. Following discontinuation of crizotinib, the aseptic inflammatory abscesses of the back gradually resolved, and the nephritic abscess also significantly reduced after drainage. Subsequently, crizotinib was resumed at a reduced dose based on clinical reassessment. During follow-up, no adverse events recurred, and the cancer remained well controlled.

According to the crizotinib prescribing information, the incidence of nephritic abscess was approximately 4% (8). Cumulative case reports suggest a potentially higher incidence among Asians compared to other ethnicities (9). In a retrospective analysis of 26 cases of crizotinib-associated nephritic abscess, Cameron et al. (10) found that 4 out of 26 cases (15.4%) were misdiagnosed as malignant, tuberculous, or other pathogen-related abscesses. Although aseptic abscesses of other organs have been documented (7), there have been no clear reports of crizotinib-induced aseptic inflammatory abscesses of the back. Given this, the condition was prone to misdiagnosis as metastatic malignancy or tuberculous abscesses, which may prolong the treatment course. In this case, the patient was initially suspected of having a tuberculosis infection: multiple tuberculosis tests were performed, all yielding negative results, while the lesion continued to expand. After excluding the tuberculous abscess, the patient subsequently underwent a surgical excision of the back abscesses. Pathology revealed the subcutaneous back abscesses to be sterile, with no pathogenic microorganisms being detected. Due to the failure to correctly diagnose crizotinib-associated aseptic inflammatory abscess, despite thorough surgical removal of the abscesses of the back, further abscess formation occurred during the incision recovery period. This ultimately led to poor wound healing and persistent abscess drainage. Therefore, early recognition and diagnosis of crizotinib-associated adverse events are critically important.

Crizotinib-associated aseptic inflammatory abscesses exhibit characteristic imaging features: CT scans typically reveal multilocular cystic lesions, with newly developed abscesses presenting enhanced capsules or septa (8, 11). MRI demonstrates T2 low signal and T1 high signal in the cyst wall, along with restricted diffusion around the abscess (12). Additionally, pathological reports (10, 13–15) of biopsy specimens from the literature typically show a fibrous capsule with chronic inflammatory infiltration, characterized by the absence of malignant tumor cells and nonspecific inflammatory features. The imaging and pathology reports of the back abscesses and nephritic abscess in this case report align with the above characteristic changes, suggesting a possible crizotinib-induced aseptic inflammatory abscesses. This prompted the multidisciplinary team to recommend temporary Crizotinib discontinuation for observation. Therefore, we recommend imaging studies as the first step in patients receiving crizotinib who are present with suspected abscesses. For patients meeting the imaging criteria, pathological biopsy should be performed. If both examinations reveal characteristic findings, a crizotinib-related aseptic inflammatory abscesses are highly probable. Further multi-case analysis should validate this approach. If confirmed, this recommendation would significantly shorten the diagnostic process.

Most crizotinib-associated inflammatory abscesses are asymptomatic. These abscesses are often present as aseptic abscesses, resolving after discontinuation of crizotinib and potentially spontaneously. In our pathology, the back abscesses were aseptic inflammatory abscesses that were resolved after discontinuation of crizotinib. However, some aseptic inflammatory abscesses may progress to complicated abscesses due to disease progression or invasive procedures, potentially leading to complications such as pain, infection, rupture, or hemorrhage (9, 16, 17). In this case report, the initial drainage fluid from the nephritic abscess exhibits a bean-paste-color, suggesting possible hemorrhage. The encapsulated hematoma likely provided an optimal environment for bacterial growth, leading to infection. Several case reports indicate that for symptomatic cases, in addition to reducing or discontinuing crizotinib, drainage of the abscesses is necessary (10, 16, 18). In this case report, the nephritic abscess was rapidly shrinked following drainage. Notably, due to the extensive size of the abscess and its proximity to the intestine, management was challenging. After discussion, staged and minimally invasive ultrasound-guided catheter drainage was employed, avoiding extensive surgical trauma. This approach enhanced comfort and reduced psychological stress of patients. Therefore, in patients with crizotinib-associated inflammatory abscesses, prompt drainage is indicated upon the onset of pain, signs of infection, or persistent symptoms after drug discontinuation. The least invasive drainage method should be prioritized based on the individual clinical scenario. Ultrasound-guided catheter drainage is the first-line option, and surgical intervention should be employed only when necessary. The mechanism by which crizotinib induces aseptic inflammatory abscesses remains unclear. Through literature review, we classified the hypotheses into three categories.: 1. Impaired tissue repair: Crizotinib is a multi-targeted tyrosine kinase inhibitor (TKI) that inhibits targets of ALK, ROS 1, and c-met proto-oncogene products (c-MET, hepatocyte growth factor receptor) (19, 20). Research suggested that this adverse event may be associated with an unknown feedback mechanism triggered by inhibition of mesenchymal-epithelial transition factor (c-MET) (18). c-MET is expressed in epithelial cells and plays a critical role in embryonic development, tissue repair, and wound healing. 2. Inflammasome activation: Tyrosine kinase inhibitors can activate NLRP3 inflammasomes in myeloid cells through lysosomal damage and cell lysis (21). The NOD-like receptor protein 3 (NLRP3) inflammasome is a protein complex that regulates innate immune responses by activating caspase-1 and inflammatory cytokines interleukin (IL)-1β and IL-18. Multiple studies indicate that NLRP3 inflammasome activation promotes the onset and progression of inflammation-related diseases (22). 3. Impaired immune cell function: Immune function in cancer patients may be suppressed, potentially leading to T-cell dysfunction under the influence of various inhibitory signals present in the tumor microenvironment (23). Additionally, patients who have undergone prior radiotherapy or chemotherapy may experience bone marrow suppression, resulting in T-cell depletion. Crizotinib exerts immunosuppressive effects on dendritic cells, diminishing their function. Collectively, these factors increase susceptibility to inflammatory infectious diseases (24). In this case report, the patient’s lymphocyte percentage remained within the normal range prior to the discovery of aseptic inflammatory abscesses. Following the identification of aseptic inflammatory abscesses, the lymphocyte percentage persistently remained below the lower limit of normal. However, after the improvement of aseptic inflammatory abscesses, the lymphocyte percentage returned to normal. This appears to support the notion that crizotinib-associated aseptic inflammatory abscesses are significantly related to impaired immune cell function. However, the underlying mechanism requires further investigation.

During the treatment of this case, we also observed persistent hypoalbuminemia and low hemoglobin levels in the patient while taking crizotinib. This finding aligns with reported hematologic adverse events associated with crizotinib (5). It suggested that multiple adverse events induced by crizotinib may coexist in a patient, necessitating comprehensive consideration and a multidisciplinary approach.

Regarding subsequent anticancer regimens, most case reports ultimately discontinued targeted therapy or switched to alternative targeted agents. For patients who initially responded well to crizotinib, the potential benefits of such adjustments warrant further investigation. Reports indicated that adjusting crizotinib dosage represents a more effective strategy for mitigating adverse events while maintaining anticancer efficacy (6, 13). In this case, subsequent blood genetic testing revealed the patient exhibited sensitivity only to crizotinib, leading to a reduced-dose regimen. Following the crizotinib dose reduction, the aseptic inflammatory abscesses ceased progression, and crizotinib-induced refractory hypoalbuminemia and anemia improved without compromising tumor control. These results not only highlight the potential of personalized therapy but also confirm the efficacy of crizotinib dose-reduction strategies in preventing lung cancer recurrence while minimizing adverse events.

Of note, although crizotinib has been superseded as first-line therapy for ALK-positive lung cancer, it remains the standard of care for ROS1-positive disease. Thus, our findings retain direct clinical relevance for ROS1-addicted patients receiving crizotinib. We hope this observation will alert oncologists to these potential adverse events in that population.

## 4 Conclusion

In summary, we report a rare manifestation in a patient with ALK-rearranged lung adenocarcinoma treated with crizotinib who developed concurrent aseptic inflammatory abscesses in the back and nephritic abscess. Early recognition and consideration of crizotinib-related adverse events are essential. For such patients, crizotinib dose reduction therapy can reduce the incidence of adverse events while maintaining effective anti-lung cancer effects. Reviewing the whole treatment, we recommend that the diagnosis of crizotinib-associated aseptic inflammatory abscesses requires the integrated interpretation of imaging, pathological features, and clinical judgment to optimize diagnostic strategies, thereby enhancing patient safety and therapeutic outcomes. Meanwhile, the treatment should be individualized, with priority given to minimally invasive approaches to avoid the risks associated with more extensive surgical interventions.

## Data Availability

The raw data supporting the conclusions of this article will be made available by the authors, without undue reservation.

## Supplementary material

Supplementary Figure 1 Follow-up abdominal computed tomography (CT) scans of the patient’s tumor. **(A)** Following crizotinib discontinuation and dose reduction, no progression of metastatic lesions in the patient’s bronchi and pulmonary nodules was observed. **(B)** Following crizotinib discontinuation and dose reduction, no progression of metastatic lesions in the patient’s liver and iliac bones was observed.
