# Supplementary material for: Aseptic inflammatory abscesses induced by crizotinib in a case report of ALK rearrangement lung adenocarcinoma
Source: Front Oncol. 2026 May 14;16:1812641. doi: 10.3389/fonc.2026.1812641 (PMC13215924; doi:10.3389/fonc.2026.1812641)
Supplement: Supplementary Figure 1 — Follow-up abdominal computed tomography (CT) scans of the patient’s tumor. (A) Following crizotinib discontinuation and dose reduction, no progression of metastatic lesions in the patient’s bronchi and pulmonary nodules was observed. (B) Following crizotinib discontinuation and dose reduction, no progression of metastatic lesions in the patient’s liver and iliac bones was observed. [file DataSheet1.pdf]

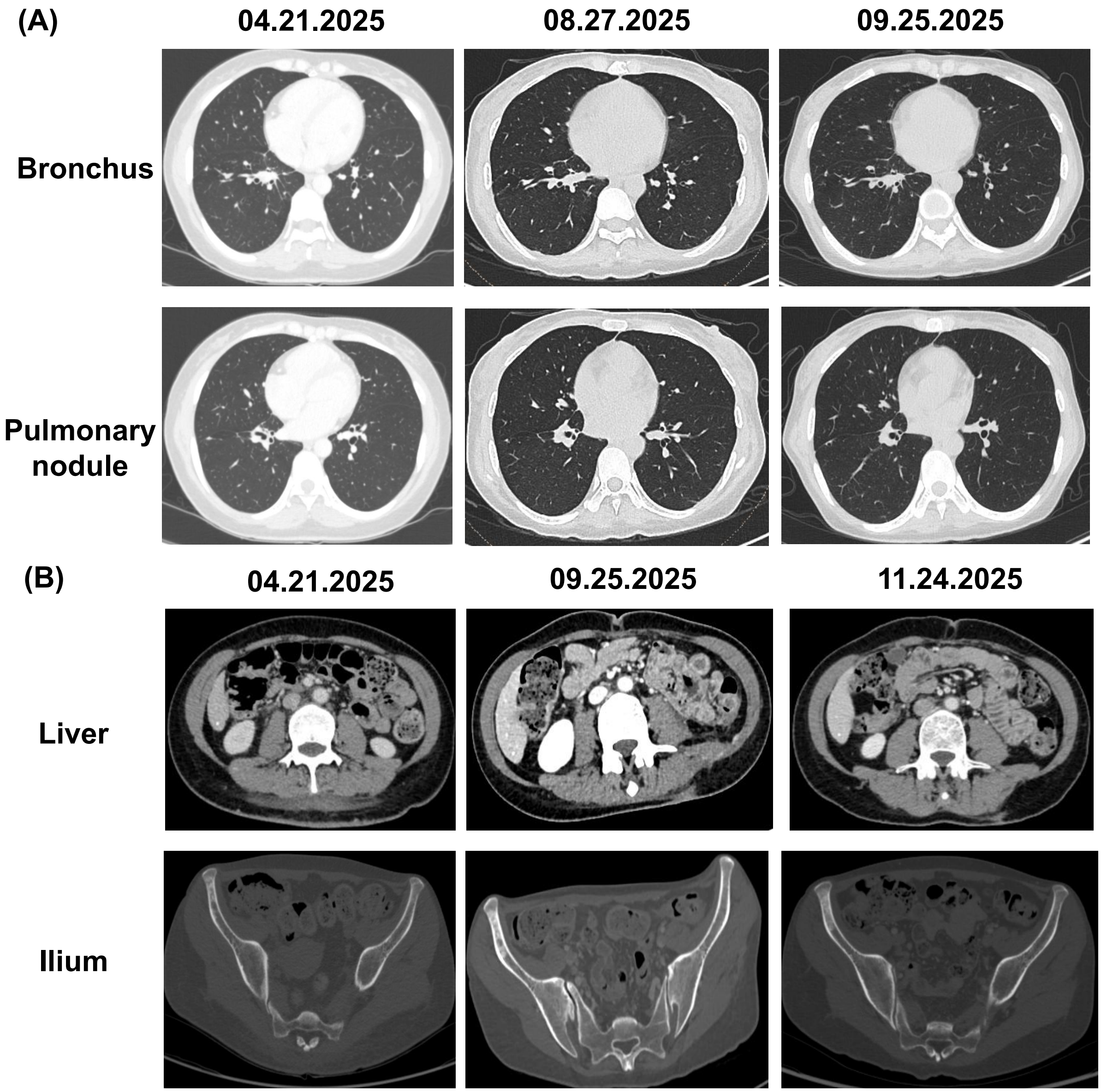

Supplementary Figure 1. Follow-up abdominal computed tomography (CT) scans of the patient's tumor (A) Following crizotinib discontinuation and dose reduction, no progression of metastatic lesions in the patient's bronchi and pulmonary nodules was observed. (B) Following crizotinib discontinuation and dose reduction, no progression of metastatic lesions in the patient's liver and iliac bones was observed.
